# Supplementary material for: Prevalence and risk factors of asymptomatic bacteriuria in community-dwelling Korean adults
Source: PLoS One. 2026 Mar 17;21(3):e0344727. doi: 10.1371/journal.pone.0344727 (PMC12994802; doi:10.1371/journal.pone.0344727)
Supplement: Attachment — Submitted filename: Response_to_Reviewers_20260109.docx [file pone.0344727.s003.docx]

**[Comment 1] Definition of ASB**

1. Response to the reviewer

Thank you for this important comment. We acknowledge that our operational definition differs from the standard IDSA/USPSTF criteria and have now explicitly stated this in the manuscript, along with justification for using urine flow cytometry–based bacteriuria (≥1+) and appropriate supporting references.

2. Location of changes in the manuscript

Methods – Outcome (Page 4, Lines 84–90)
Methods – Outcome (Page 5, Lines 91–98)
Methods – Measurements (Page 5, Lines 106–108)
Discussion – Limitations (Page 14-15, Lines 257–264)

3. Revised text

- Urine samples were collected as midstream clean-catch specimens and analyzed without centrifugation. Urinary bacteria were quantified using a UF-1000i flow cytometer (Sysmex Corp., Kobe, Japan), which detects and counts particles based on fluorescence flow cytometry using a semiconductor laser.
  The UF-1000i analyzes a defined volume of uncentrifuged urine and reports bacterial counts semi-quantitatively on a scale ranging from negative to 3+, according to the manufacturer’s predefined thresholds.
- In this study, we operationally defined bacteriuria without self-reported urgency–frequency symptoms—referred to as asymptomatic bacteriuria for the purposes of this epidemiologic analysis—as the presence of bacteriuria detected by urine flow cytometry (≥1+)* in individuals who reported no urgency–frequency symptoms, based on a negative response to the question: “Have you recently experienced any sudden and frequent need to urinate?” This operational definition is based on flow cytometry rather than quantitative urine culture and therefore differs from the Infectious Diseases Society of America (IDSA) reference definition of asymptomatic bacteriuria.

*Reference

- 1.Validation and Search of the Ideal Cut-Off of the Sysmex UF-1000i^®^ Flow Cytometer for the Diagnosis of Urinary Tract Infection in a Tertiary Hospital in Spain. *Front Med (Lausanne)*. 2018 Apr 9;5:92.
- 2. Diagnosis of Bacteriuria and Leukocyturia by Automated Flow Cytometry Compared with Urine Culture*. J Clin Microbiol*. 2010 Aug 25;48(11):3990–3996.
- Fasting blood samples (approximately 20–30 mL) and urine samples (approximately 15 mL) were collected in the morning after at least 8 h of overnight fasting.
- Second, bacteriuria without self-reported urgency–frequency symptoms was not defined using urine culture, the diagnostic gold standard, but rather by urine flow cytometry combined with the absence of self-reported urinary symptoms. Although this approach may lead to some misclassification, particularly in cases of low-grade bacteriuria or unrecognized symptoms, urine flow cytometry correlates well with conventional culture results and demonstrates high sensitivity for clinically significant bacteriuria. As such, this pragmatic approach enabled the evaluation of bacteriuria without self-reported urgency–frequency symptoms in a large community-dwelling population.

**[Comment 2] Symptom Ascertainment**

1. Response to the reviewer
We appreciate the reviewer’s concern regarding symptom ascertainment based on a single question. Because the provided dataset did not include urinary or systemic symptoms other than self-reported urgency–frequency, we were unable to further exclude individuals with potential urinary tract infection–related symptoms beyond this single item. This limitation reflects constraints of the available data rather than the study design itself. Accordingly, we have revised the Limitations section to explicitly acknowledge the potential for misclassification arising from the restricted symptom assessment.

2. Location of changes in the manuscript
Discussion – Limitations (Page 15, Lines 264–272)

3. Revised text
Third, symptom-based exclusion was conducted using a single self-reported question on urgency–frequency symptoms, as no additional urinary or systemic symptom variables were available in the dataset. Consequently, individuals with other urinary or systemic symptoms may have been misclassified as asymptomatic, while some participants with non-infectious causes of urinary frequency may have been excluded. This limitation reflects the constraints of the available data rather than the symptom screening strategy itself, and future studies incorporating a broader range of urinary and systemic symptoms are warranted to more accurately characterize bacteriuria in the general population.

**[Comment 3] Categorization of Variables**

1. Response to the reviewer
We thank the reviewer for raising concerns about variable categorization. While it was not possible to provide external justification for all cutoffs, age and sex were categorized using median values to reflect clinically meaningful groupings; in particular, the age cutoff corresponds to a period of hormonal and physiological changes in women that may influence bacteriuria epidemiology. This rationale has been clarified in the manuscript. For other variables, including serum albumin and physical activity, we conducted sensitivity analyses using continuous forms and alternative categorizations, which yielded results consistent with the primary analyses.

- Age was categorized using a cut-off of 50 years, corresponding approximately to the median age of the study population and reflecting a clinically relevant threshold associated with hormonal and physiological changes, particularly in women, which have been reported to influence the epidemiology of bacteriuria. This categorization was intended to facilitate interpretability and subgroup comparisons rather than to imply a biological dichotomy.

Reference

- Raz R; *Urinary tract infection in postmenopausal women.*; Korean Journal of Urology. 2011;52(12):801–808.
- Covariates included in the multivariate logistic regression models were selected based on prior clinical relevance and variables showing statistical significance in univariate analyses. Continuous variables were categorized using median values to facilitate clinical interpretability and subgroup comparisons. To assess the robustness of our findings, sensitivity analyses were additionally performed using continuous forms of these variables, yielding consistent results.

| Variable | Sensitivity analysis (continuous)  OR (95% CI) |
| --- | --- |
| Age (years) | 1.061 (0.890-1.264) |
| Albumin (g/L) | 0.917 (0.723-1.159) |
| Age * albumin | 0.999 (0.995-1.004) |
| Physical activity (alternative categorical) |  |
| 1 (less than 30 minutes) | 0.737 (0.403-1.240) |
| 2 (30 to less than 60 minutes) | 1.140 (0.685-1.801) |
| 3 (60 to less than 90 minutes) | 0.757 (0.353-1.425) |
| 4 (90 minutes to less than 2 hours) | 1.378 (0.685-2.512) |
| 5 (2 hours to less than 3 hours) | 1.117 (0.619-1.877) |
| 6 (3 hours to less than 4 hours) | 0.836 (0.405-1.543) |
| 7 (4 hours to less than 5 hours) | 0.560 (0.233-1.138) |
| 8 (5 hours or more) | 0.567 (0.404-0.781) |

**[Comment 4] Handling of missing data**

1. Response to the reviewer
We appreciate this comment regarding missing data. In response, we added a participant flow diagram and compared baseline characteristics between participants included in the final analysis and those excluded due to missing data to assess potential selection bias.

- Participant flow diagram

- Comparison table of included and excluded participants

| variables | Included participants (N = 8,508) | Excluded participants  (N = 1,395) | P value |
| --- | --- | --- | --- |
| Age (years) | 51.7 ± 8.8 | 55.5 ± 8.8 | < 0.001 |
| Men (%) | 4,135 (48.6) | 610 (43.7) | 0.001 |
| Current smoking (%) | 2,186 (25.7) | 351 (27.8) | 0.113 |
| Alcohol consumption (%) | 4,128 (48.5) | 520 (39.9) | < 0.001 |
| Diabetes (%) | 1,069 (12.6) | 359 (44.6) | < 0.001 |
| Chronic kidney disease (%) | 187 (2.2) | 31 (2.2) | 1.000 |
| Body mass index (kg/m^2^) | 24.6 ± 3.1 | 24.4 ± 3.2 | 0.007 |
| Serum creatinine (µmol/L) | 75.0 ± 17.4 | 72.1 ± 33.2 | 0.001 |
| Serum albumin (g/L) | 42.6 ± 3.3 | 40.8 ± 2.5 | < 0.001 |

**[Comment 5] Confounding and model specification**

1. Response to the reviewer
Thank you for this methodological comment. We have clarified our covariate selection strategy based on clinical relevance and prior literature, and assessed multicollinearity among covariates using variance inflation factors (VIFs), with all VIF values below 2.2, indicating no significant multicollinearity. Model adequacy was evaluated using goodness-of-fit measures, including the Hosmer–Lemeshow test, and no significant lack of fit was observed (Hosmer–Lemeshow χ² = 5.10, df = 8, P = 0.75). All statistical tests were two-sided, and a P value < 0.05 was considered statistically significant. Nevertheless, residual confounding due to unmeasured or incompletely measured factors cannot be entirely ruled out.

2. Location of changes in the manuscript
Statistical Analysis (Page 7, Lines 145–148)

3. Revised text
Multicollinearity among covariates was assessed using variance inflation factors (VIFs), with all VIF values below 2.2. Model adequacy was evaluated using the Hosmer–Lemeshow goodness-of-fit test, and no significant lack of fit was observed. All tests were two-sided, and a P value < 0.05 was considered statistically significant.

| Variables | VIF |
| --- | --- |
| Age | 1.23 |
| Sex | 2.11 |
| Current smoking | 1.39 |
| Alcohol consumption | 1.20 |
| College graduate | 1.04 |
| High monthly income | 1.08 |
| High physical activity | 1.03 |
| Poor self-rated health | 1.10 |
| Impaired sleep | 1.06 |
| Diabetes | 1.07 |
| Chronic kidney disease | 1.41 |
| Body mass index | 1.09 |
| Serum creatinine | 1.57 |
| Serum albumin | 1.10 |
| Hemoglobin | 1.51 |

**[Comment 6] Depth and currency of literature**

1. Response to the reviewer
We appreciate the reviewer’s suggestion to strengthen the depth and currency of the literature. In response, we revised the Discussion to incorporate contemporary clinical guidelines and recent evidence on asymptomatic bacteriuria, including updated recommendations from major organizations. In addition, several older references were replaced or supplemented with more recent studies to better reflect current understanding and clinical practice.

2. Location of changes in the manuscript
Discussion (Page 12, Lines 206–210)

Discussion (Page 14, Lines 237–251)
References list (updated to reflect revised citations)

3. Revised text

- Patients with ASB are commonly encountered in clinical practice. According to the 2019 recommendation statement by the U.S. Preventive Services Task Force, screening for and treatment of asymptomatic bacteriuria are not recommended in nonpregnant adults, as they provide no clinical benefit and may cause harm, whereas screening is recommended during pregnancy (1).

Reference

- U.S. Preventive Services Task Force Owens DK, Davidson KW, Krist AH, et al.
  Screening for Asymptomatic Bacteriuria in Adults: US Preventive Services Task Force Recommendation Statement. *JAMA.* 2019;322(12):1188–1194.
- We found that physical activity and serum albumin were independently associated with ASB, with higher levels of both factors being related to lower odds of ASB. The inverse association between physical activity and ASB is biologically plausible and may be explained by several potential mechanisms, as regular physical activity has been associated with improved insulin sensitivity and glycemic control, enhanced innate and adaptive immune responses [], and improved lower urinary tract function, which could help reduce urinary stasis and, consequently, susceptibility to bacterial colonization []. Serum albumin reflects not only nutritional status but also systemic inflammation and immune function, as it is a negative acute-phase reactant whose synthesis decreases during inflammatory states, and lower serum albumin levels have been associated with impaired host defense mechanisms and increased susceptibility to infection []. From an epidemiological perspective, serum albumin is a readily available clinical marker, and together with physical activity, these modifiable lifestyle and nutritional factors may be relevant to ASB risk beyond fixed demographic characteristics and could represent potential targets for population-level prevention strategies.

**[Comment 7] Data availability statement**

1. Response to the reviewer
Thank you for this comment. We revised the Data Availability Statement to comply with PLOS ONE policy.

2. Location

Changes were made to the Data Availability Statement file.

3. Revised text

The data underlying the results of this study are available from the Korean Genome and Epidemiology Study (KoGES), National Institute of Health. Due to ethical and legal restrictions, the data are not publicly available but can be accessed by qualified researchers through an application process administered by the National Institute of Health (http://coda.nih.go.kr). The authors did not have special access privileges and any researcher meeting the access criteria may obtain the data in the same manner.

**[Comment 8] Presentation and Clarity**

1. Response to the reviewer
We thank the reviewer for the helpful comments regarding presentation and clarity. In response, we carefully reviewed the manuscript to address minor typographical errors, improve clarity, and ensure consistency throughout the text. Specifically, figure captions and footnotes were revised, abbreviations were defined at first use, numerical formats were standardized, and all laboratory variables were expressed using consistent SI units.

2. Location of changes in the manuscript
Figure legends and footnotes (Page 7, Lines 156–159 & Page 12, Lines 196-204)

Methods – Definitions (Page 6, Lines 125–128)

Discussion (Page 13, Lines 231–233)

Minor editorial and formatting issues, including unit consistency, spacing, punctuation, and abbreviation usage, were corrected throughout the manuscript (Methods, Results, Tables, and Figure legends).

3. Revised text

- Figure 1. Prevalence of asymptomatic bacteriuria (ASB) according to age group and sex. Age groups were defined using the median age of 50 years. ASB was defined as bacteriuria detected by urine flow cytometry (≥1+) in the absence of self-reported urinary symptoms. Abbreviations: ASB, asymptomatic bacteriuria.
- Figure 2. Odds ratios (ORs) for asymptomatic bacteriuria according to sex and selected factors. ORs and 95% confidence intervals (CIs) were obtained from multivariate logistic regression models adjusted for age, sex, alcohol consumption, smoking status, education level, physical activity, monthly income, self-rated health, impaired sleep, diabetes, chronic kidney disease, body mass index, serum creatinine, serum albumin, and hemoglobin. When a variable was used for stratification, it was excluded from the corresponding regression model. Statistical significance was assessed using two-sided Wald tests. P values are indicated by symbols as follows: P < 0.001 (*), P = 0.004 (**), P = 0.010 (†), and P = 0.002 (‡). Abbreviations: OR, odds ratio; CI, confidence interval; W, women; M, men.
- The age cutoff of 50 years was chosen based on the median age of the study population and to facilitate interpretability in subgroup analyses, rather than to imply a biological threshold. As the KoGES cohort includes only adults aged 40–69 years, children and adolescents were not represented in this study.
- However, parity could not be considered due to data unavailability, which may have led to residual confounding in the association between female sex and asymptomatic bacteriuria.
