# Supplementary material for: Prevalence and risk factors of asymptomatic bacteriuria in community-dwelling Korean adults
Source: PLoS One. 2026 Mar 17;21(3):e0344727. doi: 10.1371/journal.pone.0344727 (PMC12994802; doi:10.1371/journal.pone.0344727)
Supplement: Attachment — Submitted filename: Reviewer Comments to Authors_PONE_25.pdf [file pone.0344727.s001.pdf]

Reviewer Comments to Authors

Manuscript Title: Prevalence and Risk Factors of Asymptomatic Bacteriuria in Community-Dwelling Korean Adults

1. Key Strengths

Large, well-characterized community-based cohort of 8,508 adults, enhancing statistical precision and external validity.

Clear and focused research objective addressing a regional evidence gap regarding asymptomatic bacteriuria (ASB) in Asian adults.

Use of standardized data collection and central laboratory analyses ensures consistency and reliability of biochemical measures.

Appropriate use of logistic regression analysis, including univariate and multivariable models, which supports robust inference.

Findings related to physical activity and serum albumin provide potentially novel insights that could inform future preventive studies.

Ethical approval and adherence to the Declaration of Helsinki were appropriately described, ensuring the study's ethical integrity.

2. Major Weaknesses and Points Needing Revision

- Definition of ASB:

  The definition of asymptomatic bacteriuria relies on flow cytometry with a ≥1+ threshold and the absence of a single self-reported symptom ('sudden and frequent need to urinate'). This approach deviates from standard IDSA/USPSTF definitions that depend on quantitative urine culture and broader symptom assessment. Please acknowledge this deviation clearly in the manuscript, justify the cutoff used for defining bacteriuria, and cite supporting validation studies.

- Symptom Ascertainment:

  The exclusion of symptomatic cases is based on a single question, potentially missing individuals with other urinary or systemic symptoms. Clarify the symptom screening process and expand the discussion to include possible misclassification of symptomatic individuals as asymptomatic.

- Categorization of Variables:

  The dichotomization of age (≥50 years), physical activity (≥5 hours/day), and serum albumin (≥42 g/L) appears arbitrary. Provide justification or references for these cutoffs, or consider sensitivity analyses using continuous or alternative categorical variables.

- Handling of Missing Data:

  The exclusion of 1,395 participants with missing data is not sufficiently described. Include a flow diagram illustrating participant selection and compare characteristics between included and excluded participants to assess selection bias.

- Confounding and Model Specification:

  While covariates were selected based on clinical and statistical relevance, a more explicit description of variable selection strategy is needed. Address possible multicollinearity among predictors (e.g., age, diabetes, BMI, albumin) and emphasize that observed associations may reflect residual confounding.

- Depth and Currency of Literature:

  The Discussion relies heavily on older references, with limited integration of recent ASB studies or guideline updates. Strengthen the Discussion by incorporating newer literature (e.g., post-2019 IDSA guidelines) and by situating findings within the broader global ASB research context.

- Data Availability Statement:

  The statement indicates that data are available upon request due to ethical restrictions. Ensure compliance with PLOS ONE data-sharing policies by providing the official access pathway (e.g., via KoGES/KDCA) and explaining the rationale for restricted access.

- Presentation and Clarity:

  Revise minor typographical errors (e.g., Figure 1 caption), clarify the choice of median age 50 years, and specify sample volumes for biological analyses. Ensure consistent SI units, define all abbreviations at first use, and standardize numerical formats (e.g., use periods instead of commas for decimals).
